# Varietal Descriptors for the Distinction of Underutilized Varieties of *Sechium edule* (Jacq) Swartz

**DOI:** 10.3390/plants11233309

**Published:** 2022-11-30

**Authors:** Jorge Cadena-Iñiguez, Carlos Hugo Avendaño-Arrazate, Ma. de Lourdes Arévalo-Galarza, Víctor Manuel Cisneros-Solano, Lucero del Mar Ruiz-Posadas, Juan Francisco Aguirre-Medina, Kazuo Watanabe, Ryoko Machida-Hirano, Luís Angel Barrera-Guzmán

**Affiliations:** 1 Colegio de Postgraduados, Campus San Luis Potosí, Salinas de Hidalgo, San Luis Potosi 78620, Mexico; 2 Interdisciplinary Research Group in Sechium edule in Mexico, Texcoco 56160, Mexico; 3 Instituto Nacional de Investigaciones Forestales Agrícolas y Pecuarias México, Coyoacán, Ciudad de México 04010, Mexico; 4 Colegio de Postgraduados Campus Montecillo, Texcoco 56230, Mexico; 5 Universidad Autónoma Chapingo, Centro Regional Universitario Oriente, Veracruz 94100, Mexico; 6 Facultad de Ciencias Agrícolas, Universidad Autónoma de Chiapas, Huehuetán, Chiapas 30660, Mexico; 7 Tsukuba Plant Innovation Research Center, University of Tsukuba, Tsukuba, 1-1-1 Tennodai, Tsukuba 305-8571, Ibaraki, Japan; 8 Genetic Resources Center, National Agriculture and Food Research Organization, 2-1-2 Kannondai, Tsukuba 305-8602, Ibaraki, Japan; 9 Universidad del Valle de Puebla, Calle 3 A Sur, El Cerrito, Puebla 72440, Mexico

**Keywords:** amplified fragment length polymorphism (AFLP), fruit characteristics, GenBank, molecular markers, plant variability

## Abstract

*Sechium edule* (Jacq.) Sw. (Cucurbitaceae) is a species native to Mexico and Central America. The collection, characterization, and evaluation of accessions maintained in genebanks is essential for the conservation of this species. However, there are no specific varietal descriptors that differ from those used in a phenetic approach and are adapted to international registration guidelines to help distinguish, improve, cluster, and protect intraspecific variants of common use and those obtained by breeding. Therefore, 65 morphological descriptors (qualitative and quantitative) were evaluated in 133 accessions obtained from Mexico, Guatemala, and Costa Rica located in the National Germplasm Bank of *S. edule* in Mexico. These characteristics were observed to be phenetically stable for five generations under the same agroclimatic conditions. In addition, an analysis of amplified fragment length polymorphism (AFLP) was applied to 133 samples from a set of 245 accessions. According to the multivariate analysis, 26 of the 65 descriptors evaluated (qualitative and quantitative) enabled differentiation of varieties of *S. edule*. The AFLP analysis showed a high level of polymorphism and genetic distance between cultivated accessions and their corresponding wild ancestor. The variations in *S. edule* suggest that the morphological characteristics have differentiated from an essentially derived initial edible variety (ancestral original variety), but unlike other cucurbits, there is no evidence of the ancestral edible for *Sechium* since the seed is unorthodox and there are no relicts.

## 1. Introduction

The characterization and intellectual protection of vegetables, with the aim of ex situ conservation, basic research, and genetic improvement programs, are established using a series of morphological, chemical, genetic, and physiological descriptors that fulfill the requirements of the distinction, uniformity, and stability (DUS) test, by applying the standards of the International Union for the Protection of New Varieties of Plants (UPOV), and, recently, using digital phenotyping to discriminate varietal groups [1,2]. According to UPOV [3], “a variety will be considered distinct if it differs from any other variety whose existence is commonly known, and it may be considered homogeneous if it is stable enough in its essential features, considering foreseeable variations depending on the means of reproduction, multiplication, or propagation”. Species generate varietal complexes throughout their life history; however, only a subset of the observed phenotypic variations is useful for distinguishing species and genotype. In most cases, these are species with strong traits of domestication; however, in *Sechium,* the differentiation process is related to adaptive specialization to the environment with a continuous gradient in characteristic values. Thus, traits that effectively reflect genetic differences between individuals, populations, and species are needed to accurately discern phylogenetic relationships.

Chayote (*Sechium edule (Jacq.) Sw.*) (Cucurbitaceae) is a species originally from Middle America that has been cultivated for human consumption and transferred to different agroclimatic environments. *S. edule* is a perennial ascending shrub, presenting tendrils and tuberous roots. The leaves are simple with a long petiole and lobulated or angular palmate shapes. The flowers are unisexual, axillar, staminate, and pistillate in the same knot. The fruit is pendulous, large, obovoid or piriform, with longitudinal depressions. The fruit surface is soft, light or dark green, shiny, and can be boldly or finely pubescent, with a variable number of spines, and there is a single seed [4,5].

Research projects have provided detailed descriptions of the distinctive characteristics of fruit, leaves, flowers, petioles, venation, and vines, in addition to considering the structural, biochemical, and genetic variables of *S. edule* accessions from Mexico [6].

Previous studies [7,8] reported distinctness, homogeneity, and stability to discriminate chayote varieties, and parsimony from phenetic records suggested that stable and heritable characteristics distinguished the varieties. These characteristics (classification by qualities of morphological similarity) provided the first version of possible descriptors for *S. edule* and were applied to obtain the variety *virens levis* [9]. However, these distinctive traits were very general (phenetic) and it was unknown which ones were sufficient to distinguish chayote varieties and could be generally applied with statistical validity.

The Interdisciplinary Group of Research in *S. edule* in Mexico (GISeM) has integrated different accessions of *S. edule* from Mexico, Guatemala, and Costa Rica with the highest variability in order to establish the chayote Germplasm National Bank (BANGE*Se*) in Veracruz, Mexico, where distinctive morphological descriptors were designed for validation The purpose of the research is to formalize the differentiation of genotypes using secondary characteristics, primarily morphological, that demonstrate heritability and stability in their generation, identifying the greatest genetic reciprocity with such secondary variables to facilitate their differentiation and use. Genetic diversity studies based on molecular genetic markers are widely used to distinguish genotypes of *S. edule* because, unlike many morphological traits, they are not subject to environmental influences [5,7]. Genetic diversity studies using isozymes have also been conducted [8]. However, only a few studies have examined molecular genetic diversity in chayote, and they were flawed by only including a small number of accessions and no wild progenitors [10].

The resulting morphological and biochemical characterization studies are of great importance to efficiently describe, classify, and manage accessions of plant species. Generally, the center of origin of a species date from a great genetic and morphological selection. Regarding this, Cadena et al. [11] characterized the morphological and biochemical levels of chayote accessions, where their divergences and similarities served to describe so-called chayote varietal complexes. However, chayote can also present high levels of morphological and genetic diversity in places where it has been introduced. In India, for example, high morphological and molecular diversity has been found in chayote accessions, mainly highlighting the characteristics of the fruit, and showing associations with its genomic structure based on molecular markers such as random amplified polymorphic DNA (RAPD), inter simple sequence repeats (ISSR), and directed amplification of DNA minisatellite region (DAMD) [10,12]. Primers are important tools used to describe the genetic scarcity in a species; however, the elaboration of primers requires genomic sequencing information for the species of interest. Cui et al. [13] sequenced the complete chloroplast genome for *S. edule*, opening the possibility of creating new primers for genetic studies of this species.

Molecular markers can be used to efficiently distinguish between closely related individuals of chayote. Machida-Hirano et al. [14] reported new microsatellite markers in chayote, and the obtained indicators of genetic diversity were equivalent to those obtained with analogous markers based on P450. 

Amplified restriction fragment length polymorphism (AFLP), a molecular technique used to investigate population genetics, genome mapping, relationships between molecular polymorphism, etc., can be used to analyze genetic relationships among accessions [15]. This is critical because chayote is an important vegetable in Mexico, Central America, and the Caribbean. Differentiation in biological complexes of the species may result in morphotypes with tangible values that give them local importance; however, this may also lead to identifying new uses via bioprospecting studies [16].

Many neotropical species, such as *Zea mays*, *Cucurbita* spp., and *Phaseolus vulgaris*, have been classified intraspecifically as distinctive races and ecotypes based on secondary, stable, and heritable morphological characteristics, which has facilitated their identification, conservation, and enabled new uses. The aim of this study was to evaluate the morphological characteristics and level of polymorphism of biological variants of *S. edule* and validate distinctive varietal descriptors adapted to international registration guidelines to help distinguish, improve, group, and register intraspecific varieties of common use and those obtained by plant breeding.

## 2. Results

### 2.1. Qualitative Characteristics 

The analysis of correspondence demonstrated five principal dimensions (DIM) for the qualitative variables that explained 70.43% of the variability (19.72, 9.50, 8.32, 7.64, 7.12, 5.54, 4.72, 4.39, and 3.48), and their contributions were explained in three main dimensions (Table 1). Variables that contributed to explaining the variation were DM1: VCMS (vine color at mature stage), LS (leaf shape), TC (tendril color), PC (petal color-pistillate flower), PiC (pistil color), PCSF (petal color-staminate flower), FC (fruit color), and MC (mesocarp color); DM2: LPC (leaf petiole color), PSF (presence of spines on fruit), SDF (spine density on fruit), SD (spine distribution), and SF (seed flavor); DM3: FS (fruit shape) and RF (ridges on fruit).

The analysis of hierarchical conglomerates denoted the distribution of evaluated accessions based on the qualitative characteristics and attempted to determine their relative importance as explanatory variables. In this sense, the semi-partial correlations defined the role played by each characteristic, aside from their apparent prominence in the phenotype, by means of grouping and assigned them a causality. 

**Figure 1:**
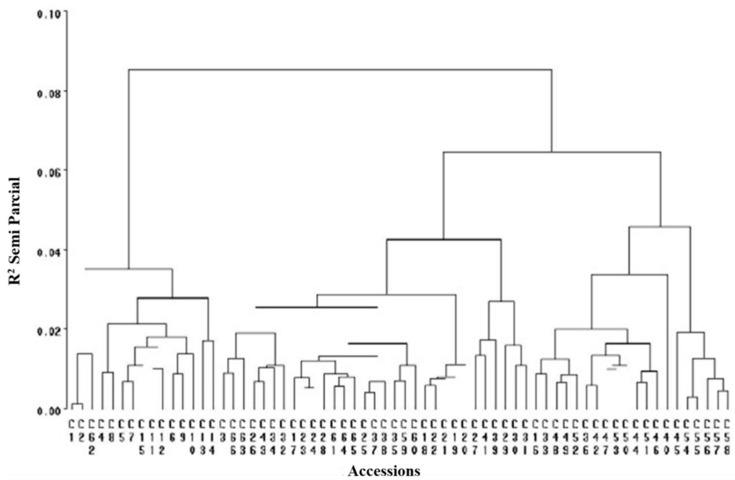
Grouping of *Sechium edule* accessions evaluated by qualitative characteristics.

Figure 1 shows the formation of a main group encoding a value of 0.09, which suggested a high similarity index between accessions for the evaluated characteristics. Different subgroups (0.06, 0.04, and 0.04) were derived from the main group, generating a series of intermediate clusters and, within them, most of the varietal groups in the inferior interval were estimated between values of 0.021 and 0.018. Qualitative variables, by their ordinal nature and considering the characteristic of distinction, avoid subjectivity and facilitate interpretation and classification because uniform intervals between measurements are unnecessary (e.g., small, medium, high, higher, or light, moderate, heavy). However, quantitative variables may acquire any value within a specified interval of values, such as dimensions of the fruit, for example, allowing that there may always be a value between any two values. Therefore, quantitative variables cannot be subjected to a criterion of order, such as variations of fruit color.

The above mentioned is relevant because of its statistical value. The outstanding variables were demonstrated to be characteristics that may be influenced by the environment; however, they continued to be important even though they could be quantitatively transformed (e.g., color, shape, spines, and dimensions), since they acquired continuity and stability in their values. Another important aspect of these variables was their relative value in the statistical load of the component, which allowed the establishment of relationships with other evaluated parameters, for example, pubescence, venation order, shape, furrows, or ribs, thus increasing the degree of continuity despite environmental influence. These variables showed unalterable characteristics that were homogeneous and uniform from generation to generation.

The values obtained from the annual measurement of morphological characteristics were lower since the standard error was very low. These results were due to measurements being taken in the month of June (beginning of flowering) and ending in late July in order to reduce crossbreeding and obtain self-pollinated samples. Some varieties began to bear fruit before emitting lateral vines (precocity), making it easier to obtain samples without interbreeding.

### 2.2. Quantitative Characteristics 

Regarding the quantitative variables, principal component analysis (PiCo) demonstrated that the first six components explained up to 78.6% of the variability of the accessions (45.6, 12.5, 5.9, 5.6, 4.6, and 4.4). The values obtained for Pearson’s correlation coefficients (PC) between two quantitative random variables was independent of their measurement scale, as shown in Table 2. The variables that contributed to the variation were: PC1: FSW (fruit size width), FSD (fruit size depth), MT (mesocarp thickness), SSL (seed size length), and SSW (seed size width); PC2: FS (flower size), RaL (rachis length), RaD (rachis diameter), and SSD (seed size depth); PC3: BL (bud length) and RL (receptacle length)

**Figure 2:**
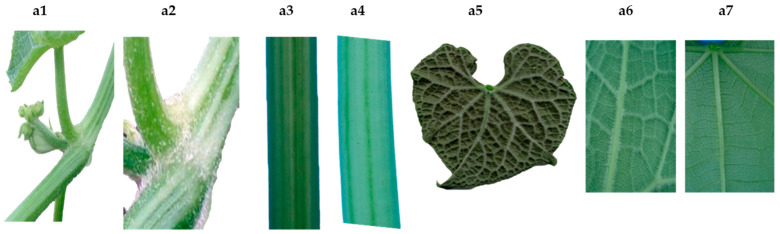
Quantitative and qualitative morphological characteristics of biological variants that allow varietal distinction of *Sechium edule*. Pubescence (**a1**,**a2**); color and striation of the guide (**a3**,**a4**); shape, pubescence, and leaf venation (**a5–a7**).

**Figure 3:**
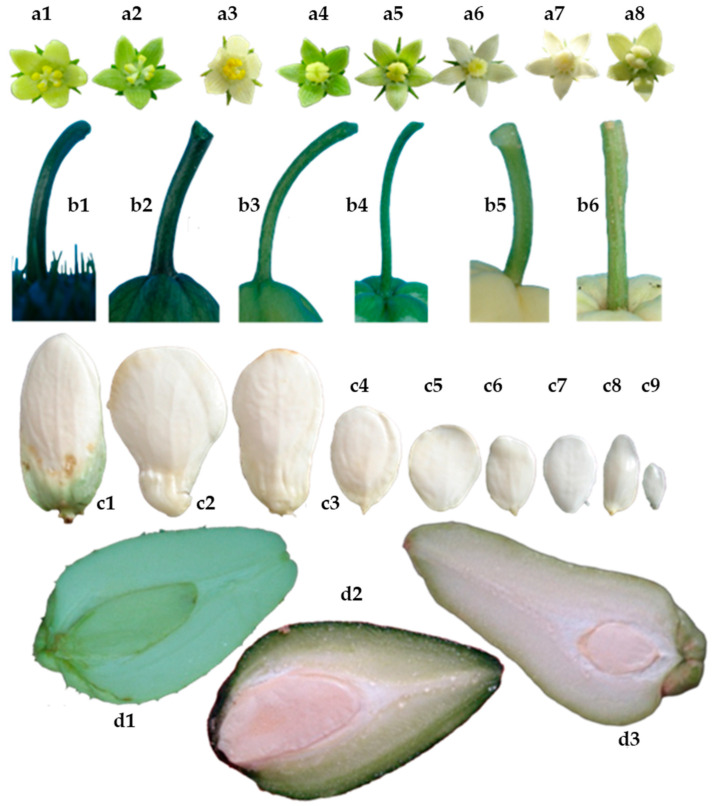
Quantitative and qualitative morphological features of biological variants that allow varietal distinction of *Sechium edule*: (**a1**–**a3**) staminate flowers, (**a4**–**a8**) pistillate flowers, (**b1**–**b6**), color, shape, thickness, and length of fruit peduncle, (**c1**–**c9**) shape, color, ornamentation, and seed length, (**d1**–**d3**) color and mesocarp thickness.

Figure 1 shows clearly defined clusters in two large groups with origins in the interval close to 0.3 and 0.4 of the correlations based on quantitative variables, indicating wide variability among these parameters reflected as two large groups with smaller subgroup formation. Based on the values from Table 2, it is assumed that a linear relationship—positive as well as negative—may exist between variables without reaching perfect dependence. The International Union for the Protection of New Varieties of Plants (UPOV) states that the distinction, uniformity, and stability (DUS) test is required to recognize a variety and thus attain legal protection. Morphological characteristics are the basis of this test, complying with the following criteria: (a) they must be clearly different from all collections of reference varieties; (b) they are maintained through different environments and times; and (c) they must be stable through reproduction. Figure 2 and Figure 3 show a wide variety of morphological characteristics that might be due to high heterogeneity in the lineage of the groups. These characteristics substantially increase the number of differences between accessions and thus a greater possibility of distinguishing varietal independence. Based in these results, Table 3 demonstrates proposed guidelines to conduct the test for distinctness, uniformity, and stability in *S. edule* (Jacq.) Sw.

### 2.3. AFLP Analysis

**Figure 4:**
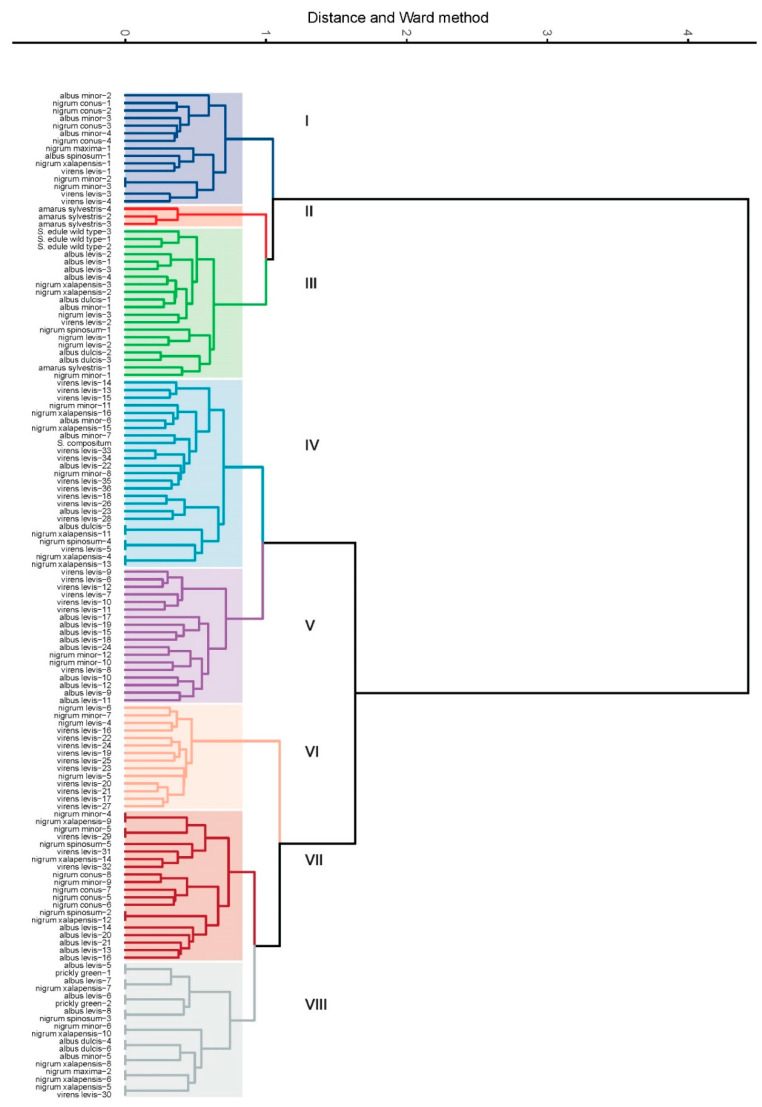
Dendrogram of accessions of *S. edule* using AFLP molecular markers, constructed using Dice coefficients and the simple hierarchical grouping method UPGMA. Roman numerals indicate the colored groups at a genetic distance of 0.215. Dendrogram of S. edule accessions using AFLP molecular markers, constructed using Dice coefficients and the UPGMA simple hierarchical clustering method. Roman numerals I–VIII indicate the groups at a genetic distance of 0.215.

In 133 individuals belonging to *S. edule*, 228 AFLP-type molecular markers were used to analyze kinship relationships between the different cultivated and wild varietal groups. Cluster analysis was run in Rstudio using the factoextra and FactoMineR packages. The distance matrix of the AFLP markers was constructed using the ade4 package and Dice coefficients. The optimal number of groups was calculated using the NbClust package, which performed a consensus of 30 indices, resulting in a majority rule, thus determining eight groups as ideal to describe the distance matrix. Group I highlighted varietal complexes with small fruit, such as *albus minor*, *nigrum conus*, and *nigrum minor*. Group II contained 75% of the *amarus sylvestris* accessions. Group III included all of the wild accessions of *S. edule.* Group IV was made up of various varietal complexes, making an in-depth discussion difficult. Groups V and VI were characterized by individuals with smooth fruit, such as *virens levis* and *albus levis*. Group VII combined the genotypes of the varietal complexes *nigrum xalapensis* and *nigrum minor*, although a subgroup with individuals of the *albus levis* varietal group was observed. Group VIII was very diverse with elements of different varietal complexes, as in groups III and IV. It was possible to make cuts in the dendrogram to create more groups with concordance, which was supported in the consulted literature and based on the varietal complexes of *S. edule* (Figure 4).

Groups I to VIII show the grouping of S. edule varieties, where certain genotypes predominated due to; however, they were not pure groups, since there are no speciation differences. They still form groups that share genetic characters even when morphologically they have differences in color, shape, size, spines, etc. This is common in groups of plants with manipulation through their life history and process of domestication and cultivation.

**Figure 5:**
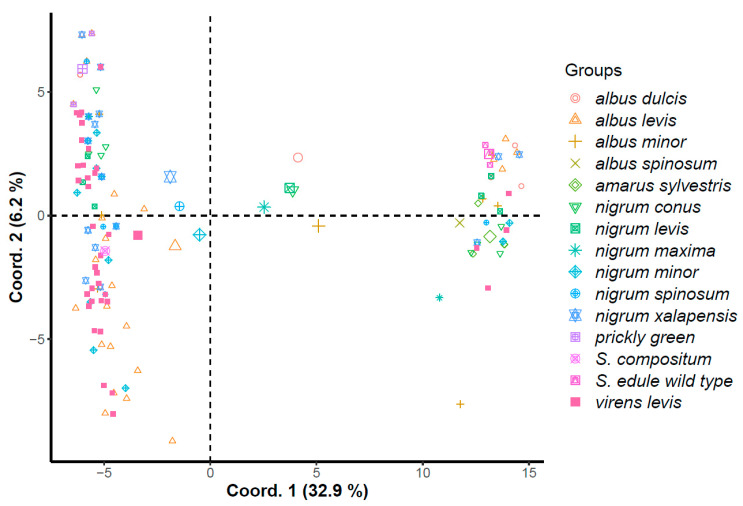
First two principal coordinates for accessions of varietal and wild complexes of *S. edule* using AFLP molecular markers.

The main coordinate analysis showed 76.58% of the total variation, in which main coordinate 1 explained 62.8% and main coordinate 2 explained 13.78%. Using the GenAlEx program, genetic distances between populations of the 133 individuals were analyzed using AFLP molecular markers. *S. edule var. amarus sylvestris* and the wild types were differentiated from the rest of the accessions, together with *S. edule var. albus spinosum*, of which only one accession was analyzed. *Sechium compositum* (included as a possible external taxon; however, this wild genotype is morphologically related to the intraspecific variations of *S. edule*, with no apparent barriers to interbreeding) clustered close to the varietal groups *virens levis, nigrum xalapensis*, and spiny green chayote. In general, the varietal complexes of *S. edule* tended to cluster in the center of the graph. However, the *nigrum levis, albus minor,* and *nigrum conus* varietal groups were located very close to each other and closest to the wild type materials because they shared common morphological characteristics (small size for *albus minor* and dark green color for *albus minor, nigrum levis,* and *nigrum conus*) (Figure 5).

## 3. Discussion

**Figure 6:**
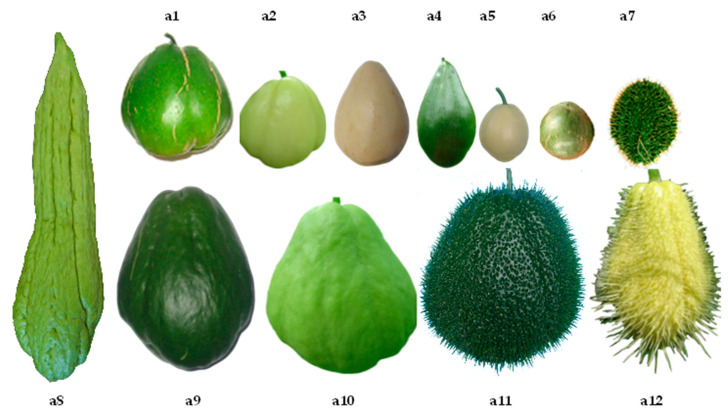
Fruit characteristics (shape, color, size, furrows, and spines) of the varieties of *Sechium edule* regarding their wild predecessor (**a7**). Medium, small, and very small fruit: (**a1**–**a6**) (*nigrum levis*, *albus levis*, *albus dulcis*, *nigrum conus*, *albus minor, nigrum minor*), big fruit (**a8**–**a12**) (*nigrum maxima, nigrum xalapensis, virens levis, nigrum spinosum, albus spinosum*) (According to [11]).

The morphological characteristics that facilitated the visual differentiation of genotypes of intraspecific complexes of *S. edule* were evident (Figure 2, Figure 3 and Figure 6), even when the correlation with genetic diversity was low, because the plants share the same origin [17,18]. However, some genotypes displayed sufficient differentiation with regards to the type closest to the wild type, suggesting that the morphological descriptors in Table 3 will allow the differentiation, organization, and identification of chayote in activities of conservation, research, and genetic improvement with greater ease of follow-up than the characteristics previously described [19,20].

Doust et al. [21] indicated that domestication, as an evolutionary process, generates changes in phenotypes, but such changes are not due to individual causes and tend to be associated with the environment, while certain phenotypic changes can take place rapidly, particularly when the histories of the populations studied are divergent. This could be the case in this study, where there were as many divergent histories as origins of the genotypes analyzed. 

Gross and Olsen [22] mentioned that both convergent phenotypical evolutions, referring to the appearance of the same trait in independent evolutional lineages, and parallel phenotypical evolution, explained as the appearance of the same trait closely related or potentially intercrossed between lineages, take place in cultivated plants. This could have happened in the varietal groups of *S. edule*. These authors related this, among other causes, to the selective introgression of a single allele through cultivation. In contrast with many cultivated plants, such as cereals in which traits that are a product of domestication have many antecedents [23], the domestication history of *S. edule* is relatively recent and mixed with environmental adaptations, generating new traits in both cases [7].

The ecological and cultural conditions of traditional agriculture have helped to preserve the genetic diversity of several economic species and influence the response of current cultivated forms to processes of empirical selection in different regions. In the case of *Sechium*, many accessions came from rural backyards with different origins. Additionally, the preferences of consumers affect the preservation of the type of chayote, such as preferences regarding the color of the fruit. For example, fruit of *var. nigrum* (dark green and very dark green) are most preferred, followed by *var. virens* (light green) and finally *var. albus* with yellow skin. This indicates an important relationship between the conservation and support of genetic resources of *S. edule* and the morphological distinctions that mark the preference of the consumer, which strongly impacts domestication or reproductive isolation.

Molecular characterization identified 91 loci with 96.7% polymorphism. The number of effective alleles was 1.18 per locus (capable of being passed on to the next generation). The expected heterozygosity had an average value of 0.127 ± 0.013, indicating moderate levels of genetic diversity compared to other species that used the same markers, such as *Zingiber officinale* (0.347) [24].

Groups of plants strongly manipulated by humans can reflect different life histories because of agroclimatic variables, reproductive isolation, and domestication processes. In this study, genetically differentiated groups were observed, some more than others, with evidence of subgrouping. Cadena-Iñiguez et al. [7] noted that a transition in the color of the fruit from dark green to light green and even to yellow was the result of adaptive specialization to the environment. Meyer et al. [25] indicated that under the domestication syndrome described by Harlan [26], changes were noted in the size of the fruit, seeds, appearance of the plant, and in secondary metabolites, among other traits of the plants. For instance, *var. amarus sylvestris* has dark green fruit and a strongly bitter flavor, while the groups *nigrum* and *virens* have fruit with a neutral flavor, and *albus* has slightly sweet fruit [11,27].

Diversity arises by speciation, forming one or more species from an ancestor [27]. Reproductive isolation induces genetic variations and, in turn, causes morphological, physiological, and chemical variations, which cause continuous (clinal) or discontinuous variations [28]. However, in *S. edule,* the soft seed coat does not allow it to be preserved [29], and there are no archaeological records that prove the existence of an initial edible variety, which suggests that those known today are essentially derived varieties. 

In northeastern India, the morphological and genetic characteristics of 74 local varieties of *S. edule* were evaluated to promote the development of breeding programs. The morphological characteristics were related to the fruit (color, length, width, and weight) and sugar, ascorbic acid, and phenol content. To complement this study, 28 RAPD-type molecular markers and 30 ISSR were added. The RAPD analysis showed a polymorphism of 88.38% and some of the chayote varieties that shared common morphological characteristics (light green and dark green fruit) also shared the same genomic fragments. Referring to the ISSR analysis, a polymorphism of 62.5% was observed. The fruit with light green and dark green tones showed the highest ascorbic acid and phenol content. 

Principal component analysis indicated that 77.74% of the total variation was explained by the first three components. The first main component was supported by the morphological characteristics of the fruit (length, width, and color), while the second component was characterized by the chemical characteristics of the fruit (ascorbic acid and total sugar content). Light green chayote varieties showed the highest average rates of polymorphism for ISSR and RAPD markers. The cluster analysis revealed the absence of a relationship between clusters and geographical distances of the chayote varieties. The results obtained, in terms of chemical characteristics, were similar to those reported by Cadena-Iñiguez et al. [11].

In 36 accessions of chayote from India, 18 quantitative and qualitative morphological characteristics of fruits were evaluated. The same study was complemented with 12 DAMD-type molecular markers. Principal component analysis showed that 49.68% of the total variation was explained by the first two components. The main characteristics that contributed to this variation were weight, shape, length, and width of the fruit, as well as length and density of the loin. These characteristics were distinctive of the chayote varietal complexes described by Cadena et al. [11]. Molecular analysis revealed 95% polymorphism, indicating high genetic diversity among chayote accessions. Unlike the study by Verma et al. [12], it was observed that cluster analysis with DAMD-type molecular markers discriminated accessions by geographical distribution (northeast and south India).

Cui et al. [13] sequenced the complete chloroplast genome of *S. edule*, which was 154,550 bp in size and contained a total of 122 genes (78 for protein coding, eight rRNA genes, and 36 tRNA genes). The guanine-cytokine content was 37.2%. The results of sequencing this genome demonstrated high phylogenetic affinity with other chloroplast genomes of species belonging to the Cucurbitaceae family, mainly *Cucumis* spp. and *Cucurbita* spp.

Our study bears similarities to other research on genetic and morphological diversity conducted in India [10,11]. The main components of the analysis indicated that the variation was linked to the morphological characteristics of the fruit (color, shape, size, and weight). On the other hand, high polymorphism was noted in the different types of molecular markers used. The associated analysis between morphological and genetic markers enriches the information regarding these accessions, thus enabling taxonomic and phylogenetic inferences.

The morphological variations observed in *S. edule* suggest that the plants have conserved the expression of essential characteristics resulting from the initial genotype or from the subsequent combination of genotypes of different varieties [30]. Figure 4 and Figure 5 indicate the genetic closeness between *var. amarus sylvestris* and the wild type (ancestor). The high level of genetic polymorphism, attributed to adaptive specialization, reproductive isolation, conservation in backyards, intercrossings, constant selection, etc., has not erased the initial characteristics, except for the bitter taste, the loss of which was attributed to cultivation, domestication, and adjustments in the mevalonic acid pathway that modified the cucurbitacin and pigment content, thus facilitating adaptation to new environments [7,11].

## 4. Materials and Methods

### 4.1. Environmental Conditions

The Germplasm National Bank of *Sechium edule* (BANGESe) is in Veracruz, Mexico (19°08′48″ N and 97°57′00″ W). The vegetation type is mountain cloud forest (altitude of 1340 m), with an annual mean temperature of 19–22 °C, relative humidity of 85–90 %, and annual mean precipitation of 2250 mm. The soils are vitric and luvisolic, rich in organic matter, low in calcium, and high in iron, manganese, and zinc nutrients, with moderate fertility, thick texture, and fragments of volcanic glass (pH 4.3–6.5) [11].

### 4.2. Biological Material

The plants used in this study have been included in BANGESe since 2005 and are native to Costa Rica, Guatemala, and the Mexican states of Chiapas, Oaxaca, Puebla, Morelos, Hidalgo, Michoacán, Nayarit, San Luis Potosí, State of Mexico, and Veracruz (Figure 6). Most of the accessions came from backyard areas and in many cases there was reproductive isolation determined by consumer preference. The sample size included 133 live accessions obtained through the choice proposed [31] from a population of 245 accessions in the germplasm bank (Supplementary Material).

Because previous biochemical, morphological, and anatomical studies showed stability of the distinctive features of *S. edule*, the number of individuals per accession [2] was reduced and the number of generations evaluated was increased to five. The successive evaluation of the accessions was carried out annually for five generations, harvesting fruit from two chosen plants per accession at physiological maturity, and the plants generated from these fruit were replanted in an open area adjacent to the Genebank. Given the previous results of parsimony and stability, the sample size (n = 133) was maintained in each generational evaluation. To reduce the risk of pollen contamination, successive fruit were selected from the first third of the plant. Multiple plants were generated and identified in order to identify the typical characteristics of the corresponding varietal group. The morphological evaluation was carried out annually for five generations, measuring each year, adhering to the protocol of the *Sechium* germplasm bank for accumulating evidence of characteristic stability. This was important since the original provenance of each accession differed.

### 4.3. Evaluation of Morphological Characteristics

The sample size for this analysis (n = 133) started from a population of 245 accessions in the germplasm bank. The agroclimatic conditions for all accessions were the same (2.0 ha of collection surface) and their essential characteristics were recorded for for five generations. Table 4 shows the morphometric patterns of the varietal descriptors designed and analyzed separately using qualitative (47) and quantitative (18) measures (UPOV). 

### 4.4. DNA Extraction and AFLP Analysis

Young and healthy leaves of the 133 chayote plant accessions were extracted to obtain the DNA using the CTAB (cetyltrimethylammonium bromide) method with some modifications [32]. Throughout the study, the same accessions were used for both morphological and AFLP analyses. Data not reported in this study indicated that the progeny of the evaluated accessions were uniformly conserved and inherited the morphological characteristics in different environments, indicating their stability.

The products from PCR were corroborated on agarose gel (0.8%) for 60 min at 80 V, and later diluted before the pre-amplification reaction was carried out. The products of the amplification were separated by polyacrylamide gel electrophoresis (6%) at 200 V for 3 h, the gels were stained with silver nitrate, and then a 1:10 dilution with formamide was carried out for the PCR-selective products, which were evaluated by capillary electrophoresis using a 3500 XL Genetic Analyzer (Thermo Fisher Scientific, Waltham, MA, USA). 

For the principal coordinate analysis, the percentage of polymorphism per band and percentage of variation explained were determined using AFLP-SURV 1.0 [33] software, and MEGA v.7 software was used to generate the tree [34]. Using Structure 2.3.4 software [35], a Bayesian analysis was carried out based on the Markov Chain Monte Carlo (MCMC) algorithm in order to assign individuals to populations based on their genotypes, estimating the frequency of population alleles and identifying their structure by explaining the presence of the Hardy-Weinberg or linkage disequilibrium [36,37].

The genetic distances were scored according to the presence (1) or absence (0) of 228 AFLP markers and the Dice coefficients [38]. Construction of the dendogram, generated using the hierarchic clustering method with the unweighted pair group method using arithmetic averages (UPGMA) [39], and principal coordinate analysis were performed using the GenAlEx 6.0 program [40].

## 5. Conclusions

Twenty-six varietal descriptors allowed differentiation between the evaluated chayote accessions. The main characteristics that enabled distinction were considered qualitative and quantitative; however, the evaluations under field conditions over five years demonstrated that the features were stable and unalterable from generation to generation. The visual distinction manifested mainly in fruit variables, such as color, shape, size, flavor, and presence/absence of spines. There was a high level of genetic polymorphism between cultivated accessions and their wild ancestor. The polymorphic variations in *S. edule* suggest that the morphological characteristics have been differentiating from an essentially derived ancestral variant. For this reason, we suggest that the characteristics evaluated in the current study should be adopted as the main varietal descriptors in the technical guide of this phytogenic resource for food and agriculture. These descriptors will update the initial guide (phenetics) for the distinction, registration, and legal protection of varieties, both those of common use and those obtained by breeding. They will also contribute to varietal separation and differentiation for ex situ conservation purposes, basic research, and bioprospective studies in a format suitable for international use.

## Figures and Tables

**Table 1:** Characteristic values and their relative and absolute contribution to the first three principal dimensions of the analysis of variability of *Sechium edule*.

	Relative Contribution			Absolute Contribution		
	Dim1 ^z^	Dim2	Dim3	Dim1	Dim2	Dim3
VCYS	0.0001	0.0018	0	0.0025	0.0213	0.0002
VCMS	0.0972	0.0231	0.0292	0.3864	0.0442	0.0489
VS	0.0004	0	0	0.0461	0.0008	0
SC	0.0471	0.0007	0.0058	0.4846	0.0036	0.0254
BP	0.0049	0	0.0007	0.1805	0.0008	0.0102
NP	0.0038	0.0009	0	0.0382	0.0045	0
**LS**	0.1503	0.041	0.0369	0.372	0.0489	0.0385
LC	0.0014	0.001	0.0056	0.0302	0.0103	0.049
LAP	0.0084	0.024	0	0.0868	0.1197	0
LVT	0.0034	0.0006	0.0001	0.1249	0.0102	0.0013
VC	0.0051	0.0028	0.003	0.2427	0.0654	0.0609
VO	0.0001	0.0001	0.0007	0.0026	0.0013	0.0116
**LPC**	0.0034	0.1102	0.0032	0.0156	0.2463	0.0062
PP	0.0018	0.0021	0.0001	0.2365	0.1292	0.0029
PD	0.0037	0.0098	0	0.0498	0.0632	0.0001
LPS	0.0008	0.0007	0	0.2173	0.0918	0.0008
**TC**	0.042	0.0115	0.0055	0.2739	0.036	0.0151
TB	0.0004	0	0	0.0286	0.0003	0.0013
TP	0.0016	0.0014	0	0.2173	0.0918	0.0008
**PC**	0.0834	0.0063	0.0015	0.4153	0.0151	0.0032
CC	0.0021	0	0.0008	0.0286	0.0001	0.0048
RC	0.0398	0.0077	0.0257	0.2259	0.021	0.0616
RP	0	0.0466	0.0063	0.0003	0.2035	0.0241
**PiC**	0.0077	0.0014	0.0048	0.1703	0.0145	0.0441
PFPB	0.0064	0.0014	0.0058	0.118	0.0125	0.0447
**PCSF**	0.1217	0.0114	0.0224	0.4765	0.0215	0.037
RS	0.0008	0.0007	0	0.2173	0.0918	0.0008
RPV	0.0001	0.0017	0.0059	0.0027	0.0223	0.0665
TEC	0.0088	0.0042	0.0005	0.1721	0.0397	0.0038
**FC**	0.0913	0	0.0586	0.3621	0	0.098
**FS**	0.0144	0.1028	0.0996	0.0602	0.2076	0.1761
**PSF**	0.0041	0.0476	0.0024	0.072	0.4067	0.0176
**SDF**	0.0011	0.1141	0.0053	0.0097	0.5091	0.0208
**RF**	0.0881	0.0009	0.5179	0.2258	0.0011	0.5597
**SD**	0.0121	0.1737	0.0141	0.0491	0.3379	0.024
PCO	0.0625	0.0086	0.0544	0.4219	0.0279	0.1549
PEP	0.0024	0.0057	0.0005	0.039	0.0452	0.0032
BGP	0.0018	0.0065	0.0103	0.0569	0.0985	0.136
**MC**	0.0511	0.0027	0.0404	0.2691	0.0068	0.0896
PF	0.0011	0.0008	0.0001	0.0836	0.03	0.0038
AFS	0.0002	0.0004	0.0008	0.0033	0.0039	0.0064
RFF	0.0034	0.0009	0.0026	0.0624	0.0079	0.0196
SC	0.0074	0.0034	0.0059	0.1307	0.0292	0.0441
SS	0.0003	0.054	0.0111	0.0012	0.111	0.0199
SO	0.0065	0.005	0.0016	0.1778	0.0661	0.0189
**SF**	0.0035	0.1568	0.0027	0.0177	0.3771	0.0056
VI	0.0018	0.0022	0.0016	0.1098	0.0635	0.0405

^z^. Dimension.

**Table 2:** Pearson’s correlation coefficients for the first three main components of quantitative variables in the analysis of variability of *Sechium edule*.

				Pearson Correlation		
	PC1	PC2	PC3	PC1	PC2	PC3
BL	0.119	0.187	0.324	0.0018	0.0112	0.0024
NML	0.171	0.138	−0.010	<0.0001	0.0641	0.9292
LPL	0.219	0.091	0.139	<0.0001	0.2244	0.2075
LPD	0.221	0.053	0.096	<0.0001	0.4822	0.3848
LMT	0.228	0.206	0.089	<0.0001	0.0051	0.4189
RL	0.137	0.020	0.464	0.0003	0.7897	<0.0001
FS	0.212	0.268	−0.313	<0.0001	0.0002	0.0034
PL	0.206	0.243	−0.197	<0.0001	0.0008	0.0713
RaL	0.145	0.394	0.211	0.0001	<0.0001	0.0524
RaD	0.171	0.257	−0.011	<0.0001	0.0004	0.9231
FSL	0.210	−0.146	0.278	<0.0001	0.0501	0.0098
FSW	0.265	−0.093	−0.119	<0.0001	0.2149	0.2787
FSD	0.292	−0.142	−0.045	<0.0001	0.0572	0.6851
PLF	0.210	0.142	−0.175	<0.0001	0.0562	0.1095
MT	0.293	−0.099	−0.006	<0.0001	0.1887	0.9557
SSL	0.238	−0.323	−0.004	<0.0001	<0.0001	0.9687
SSW	0.266	−0.246	−0.188	<0.0001	0.0007	0.0856
SSD	0.171	−0.360	−0.154	<0.0001	<0.0001	0.1599

**Table 3:** Proposed descriptors to carry out the test for distinction, uniformity, and stability (DHE) in underutilized varieties of *Sechium edule*.

Code	Descriptor-Characteristic	Description	Status
VCMS	Vine color at mature stage	Dark green with brown stripe, green, light green with brown stripe, yellow with brown stripe	1,2,3,4
LS	Leaf shape	Angular, cordiform, palmately lobed, tripartite, deltoid, sectioned	1,2,3,4,5,6
LPC	Leaf petiole color	White, light green, green, dark green, very dark green	1,2,3,4,5
TC	Tendril color	Light green, green, dark green	1,2,3
PC	Petal flower color (pistillate flower)	White, green, yellow green	1,5,9
PCSF	Petal color (staminate flower)	White, green, yellow green	1,5,9
PiC	Pistil color	White, green, yellow green	1,5,9
FC	Fruit color	White, yellow cream, light green, green, dark green	1,2,3,4,5
FS	Fruit shape	Conical, pyriform, spheroid, ovoid, cylindrical, obovoid, wide obovoid, ellipsoid, wide ellipsoid	1,2,3,4,5,6,7,8,9
FSW	Fruit size (width cm)	Very small, small, medium, long, very long	1,3,5,7,9
FSD	Fruit size (depth cm)	Very small, small, medium, long, very long	1,3,5,7,9
RF	Ridges on fruit	Absent, present	1,9
PSF	Presence of spines on fruit	Absent, present	1,9
SDF	Spine density on fruit	Very few, few, medium, many	1,3,5,7
SD	Spine distribution	Very few, few, medium, many	1,3,5,7
MC	Mesocarp color (pulp)	White, yellow cream, light green, green, dark green	1,2,3,4,5
MT	Mesocarp thickness (cm)	Thin, medium, thick	3,5,7
SF	Seed flavor	Neutral, sweet, bitter	1,3,5
SSL	Seed size (length cm)	Short, medium, long	3,5,7
SSW	Seed size (width)	Short, medium, long	3,5,7
SSD	Seed size depth	Small, medium, long	3,5,7
BL	Bud length (cm)	Short, medium, long	3,5,7
RL	Receptacle length (mm)	Short, medium, long	3,5,7
RaL	Rachis length (cm)	Short, medium, long	3,5,7
RaD	Rachis diameter (mm)	Very small, small, medium, long	1,3,5,7
FS	Flower size (length from petal to petal, cross measured) (mm)	Small, medium, long	3,5,7

**Table 4:** Quantitative characteristics of evaluation, referring to distinction, uniformity, and stability (DUS) of the chayote plant (*Sechium edule*) variants according to UPOV.

	Qualitative				Quantitative
Code	Character	Code	Character	Code	Character
VCYS	Vine color at young stage	PCSF	Pistillate flower per bud	BL	Bud length (cm)
VCMS	Vine color at mature stage	PCSF	Petal color (staminate flower)	NML	Number of mucronate leaves
VS	Vine striation	RS	Rachis shape	LPL	Leaf petiole length (cm)
SC	Striation color	RPV	Rachis pubescence	LPD	Leaf petiole diameter (cm)
BP	Bud pubescence	TEC	Teak color	LMT	Length of main tendril (cm)
NP	Nude pubescence	FC	Fruit color	RL	Receptacle length (mm)
LS	Leaf shape	FS	Fruit shape	FS	Flower size (length from petal to petal, cross measured) (mm)
LC	Leaf color	PSF	Presence of spines on fruit	PL	Pistil length (mm)
LAP	Leaf abaxial pubescence	SDF	Spine density on fruit	RaL	Rachis length (cm)
LVT	Leaf venation type	RF	Ridges on fruit	RaD	Rachis diameter (mm)
VC	Venation color	SD	Spine distribution	FSL	Fruit size (length cm)
VO	Venation order	PCO	Peduncle color	FSW	Fruit size (width cm)
LPC	Leaf petiole color	PEP	Peduncle pubescence	FSD	Fruit size (depth cm)
PP	Petiole pubescence	BGP	Basal groove presence	PLF	Peduncle length (cm)
PD	Petiole depression	MC	Mesocarp color (pulp)	MT	Mesocarp thickness (cm)
LPS	Leaf petiole shape	PF	Presence of fibers	SSL	Seed size (length cm)
TC	Tendril color	AFS	Adhesion of fiber to seed	SSW	Seed size (width cm)
TB	Tendril branching (ramification)	RFF	Raw fruit flavor	SSD	Seed size (depth cm)
TP	Tendril pubescence	SC	Seed color		
PC	Petal color (pistillate flower)	SS	Seed shape		
CC	Chalice color	SO	Seed ornamentation		
RC	Receptacle color	SF	Seed flavor		
RP	Receptacle pubescence	VI	Viviparity		
PiC	Pistil color				

## Data Availability

This research has uploaded to the Plants platform, the supplementary material related to the passport database of the genotypes evaluated.

## Supplementary Materials

The following are available online at https://www.mdpi.com/article/10.3390/plants11233309/s1.

## References

[1] Montoya-Aramburu M.A., Rodríguez N., Pérez-Almeida I., Marín C. (2008). Identificación de descriptores morfológicos relevantes para la distinción de variedades y líneas élites de arroz venezolano con fines de protección intelectual. Agron. Trop..

[2] Pereira-Dias L., Fita A., Vilanova S., Sánchez-López E., Rodríguez-Burruezo A. (2020). Phenomics of elite heirlooms of peppers (*Capsicum annuum* L.) from the Spanish centre of diversity: Conventional and high-throughput digital tools towards varietal typification. Sci. Hort..

[3] UPOV (2004). Arroz (Oryza sativa L.). Directrices Para la Ejecución del Examen de la Distinción, la Homogeneidad y la Estabilidad.

[4] Barrera-Guzma L.A., Cadena-Iñiguez J., Legaria-Solano J.P., Sahagun-Castellanos J. (2021). Phylogenetics of the genus Sechium P. Brown: A review. Spanish J. Agric. Res..

[5] Lira-Saade R. (1996). Chayote. Sechium edule (Jacq.) Sw.

[6] Cadena-Iñiguez J., Arévalo-Galarza M.L., Avendaño-Arrazate C.H., Soto-Hernández M., Ruiz-Posadas L.M., Santiago-Osorio E., Acosta-Ramos M., Cisneros-Solano V.M., Aguirre-Medina J.F., Ochoa-Martínez D. (2007). Production, Genetics, Postharvest Management and Pharmacological Characteristics of *Sechium edule* (Jacq.) Sw. Fresh Prod. Global Sci..

[7] Cadena-Iñiguez J., Avendaño-Arrazate C.H., Soto-Hernández M., Ruiz-Posadas L.M., Aguirre-Medina J.F., Arévalo-Galarza M.L. (2008). Infraspecific variation of *Sechium edule* (Jacq.) Sw. in the state of Veracruz, Mexico. Gen. Res. Crop Evol..

[8] Avendaño-Arrazate C.H., Cadena-Iñiguez J., Arévalo-Galarza M.L., Cisneros-Solano V.M., Aguirre-Medina J.F., Moreno-Pérez E.C., Cortés-Cruz M., Castillo-Martínez C.R., Ramírez-Vallejo P. (2012). Genetic variation of an infraspecific chayote complex evaluated by isoenzimatic systems. Pesq. Agrop. Bras..

[9] Cadena-Iñiguez J., Avendaño-Arrazate C.H., Cisneros-Solano V.M., Ruiz-Posadas L.M., Arévalo-Galarza M.L., Aguirre-Medina J.F. (2016). Protección de variedades criollas de uso común de chayotes Mexicanos. Agroproductividad.

[10] Jain J.R., Timsina B., Satyan K.B., Manohar S.H. (2017). A comparative assessment of morphological and molecular diversity among *Sechium edule* (Jacq.) Sw. accessions in India. 3 Biotech.

[11] Cadena-Iñiguez J., Soto-Hernández M., Arévalo-Galarza L., Avendaño-Arrazate C., Aguirre Medina J., Ruiz-Posadas L. (2011). Biochemical Characterization of Domesticated Varieties of *Chayote Sechium Edule* (Jacq.) Sw. Fruits Compared to Wild Relatives. Rev. Chapingo Ser. Hortic..

[12] Verma V.K., Pandey A., Jha A.K., Ngachan S.V. (2017). Genetic Characterization of Chayote [Sechium Edule (Jacq.) Swartz.] Landraces of North Eastern Hills of India and Conservation Measure. Physiol. Mol. Biol. Plants.

[13] Cui H., Zhu Z., Lu Z., Ding Z., Zhang C., Luan F. (2021). The Complete Chloroplast Genome Sequence of the Sechium Edule (Jacq.) Swartz. (*Cucurbitaceae*). Mitochondrial DNA Part B.

[14] Machida-Hirano R., Cortés-Cruz M., Amaro-González B.A., Cadena-Íñiguez J., Shirata K., Watanabe K.N. (2015). Isolation, and characterization of novel microsatellite markers in chayote (*Sechium edule* (Jacq.) Sw.). Am. J. Plant Sci..

[15] Wooten J.A., Tolley-Jordan L.R. (2009). Validation of phylogenetic signals in amplified fragment length data: Testing the utility and reliability in closely related taxa. BMC Res. Not..

[16] Iñiguez-Luna M.I., Cadena-Iñiguez J., Soto-Hernández R.M., Morales-Flores F.J., Cortes-Cruz M., Watanabe K.N., Machida-Hirano R., Cadena-Zamudio J.D. (2021). Bioprospecting of *Sechium* spp. varieties for the selection of characters with pharmacological activity. Sci Rep..

[17] Gethi J.G., Labate J.A., Lamkey K.R., Smith M.E., Kresovich S. (2002). SSR Variation in important U.S. maizes inbred lines. Crop Sci..

[18] Romero-Severson J., Smith J.S.C., Ziegler J., Hauser J., Hookstra G. (2001). Pedigree analysis and heliotype sharing within diverse groups of *Zea mays* L. Inbreeds. Theor. Appl. Genet..

[19] Sosa M., Galarza J.L., Lebgue T. (2006). Clasificación de las comunidades vegetales en la Región Árida del Estado de Chihuahua, México. Ecol. Apl..

[20] Khadivi A. (2018). Phenotypic characterization of *Elaeagnus angustifolia* using multivariate analysis. Ind. Crops Prod..

[21] Doust A.N., Lukens L., Olsen K.M., Mauro-Herrera M., Meyer A., Rogers K. (2013). Beyond the single gene: How epistasis and gene-by environment effects influence crop domestication. Proc. Nat. Acad. Sci. USA.

[22] Gross L.B., Olsen M.K. (2010). Genetic perspectives on crop domestication. Trends Plant Sci..

[23] Doebley J., Stec A., Hubbard L. (1997). The evolution of apical dominance in maize. Nature.

[24] Jatoi S.A., Kikuchi A., Ahmad D., Watanabe K.N. (2010). Characterization of the genetic structure of mango ginger (*Curcuma amada* Roxb.) from Myanmar in farm and genebank collection by the neutral and functional genomic markers. Electron. J. Biotechnol..

[25] Meyer R.S., Ashley E., DuVal A.E., Jensen H.J. (2012). Patterns and processes in crop domestication: An historical review and quantitative analysis of 203 global food crops. New Phytol..

[26] Harlan J.R., Anderson P.C. (1992). Wild grass seed harvesting and implications for domestication. Prehistoire de L’agricuture: Nouvelles Approches Experimentales et Ethnographiques.

[27] Rabosky D.L. (2016). Reproductive isolation and the causes of speciation rate variation in nature. Biol. J. Linn. Soc..

[28] Achigan-Dako E.G., Avohou E.S., Linsoussi C., Ahanchede A., Vodouhe R.S., Blattener F.R. (2015). Phenetic characterization of *Citrullus* spp. (Cucurbitaceae) and differentiation of egusi-type (*C. mucosospermus*). Genet. Resour. Crop Evol..

[29] Ramírez-Rodas Y., Arévalo-Galarza M.L., Cadena-Iñiguez J., Delgado-Alvarado A., Ruiz-Posadas L., Soto-Hernández M. (2021). Postharvest storage of three chayote (*Sechium edule* (Jacq.) Sw.) varieties. Sci. Agrop..

[30] UPOV (2009). General Introduction to the Examination of Distinctness, Uniformity and Stability and the Development of Harmonized Descriptions of New Varieties of Plants.

[31] Trejo-Téllez B.I., Morales-Flores F.J. (2009). Manual Para la Elaboración de Una Encuesta Rural.

[32] Turaki A.A., Ahmad B., Maaji U.F., Abdulrazak U.K., Yusuf B.A., Hamza A.B. (2017). Optimised cetyltrimethylammonium bromide (CTAB)DNA extraction method of plant leaf with high polysaccharide and polyphenolic compounds for downstream reliable molecular analyses. Afr. J. Biotechnol..

[33] Vekemans X., Beauwens T., Lemaire M., Roldan-Ruiz I. (2002). Data from amplified fragment length polymorphism (AFLP) markers show indication of size homoplasy and of a relationship between degree of homoplasy and fragment size. Mol. Ecol..

[34] Kumar S., Stecher G.G., Tamura K. (2016). MEGA7: Molecular evolutionary genetics analysis version 7.0 for bigger datasets. Mol. Biol. Evol..

[35] Pritchard J.K., Stephens M., Donnelly P. (2000). Inference of population structure using multilocus genotype data. Genetics.

[36] Falush D., Stephens M., Pritchard J.K. (2007). Inference of population structure using multilocus genotype data: Dominant markers and null alleles. Mol. Ecol. Not..

[37] Hubisz M.J., Falush D., Stephens M., Pritchard J.K. (2009). Inferring weak population structure with the assistance of sample group information. Mol. Ecol. Res..

[38] Rohlf F.J. (2000). NTSYS-pc: Numerical Taxonomy and Multivariate Analysis System, Version 2.1.

[39] Addinsoft A. XLSTAT Statistical and Data Analysis Solution. 2020, Long Island, NY, USA. https://scholar.google.es/scholar?q=XLSTAT+statistical+and+data+analysis+solution.&hl=es&as_sdt=0,5#.

[40] Peakall R., Smouse P.E. (2012). GENEALEX 6. Genetic analysis in Excel. Population genetic software for teaching and research update. Bioinformatics.

